# Elderly Mortality due to Ambulatory Care Sensitive Conditions and Primary Health Care Coverage in the Federal District

**DOI:** 10.1590/0034-7167-2022-0170

**Published:** 2022-12-16

**Authors:** Geraldo Marques da Costa, Helena Eri Shimizu, Mauro Niskier Sanchez

**Affiliations:** I Universidade de Brasília. Brasília, Distrito Federal, Brazil

**Keywords:** Aged, Primary Health Care, Mortality, Aging, Family Health, Anciano, Atención Primaria de Salud, Mortalidad, Envejecimiento, Salud de la Familia, Idoso, Atenção Primária à, Saúde, Mortalidade, Envelhecimento, Saúde da Família

## Abstract

**Objectives:**

To describe the mortality coefficients of elderly due to primary care sensitive conditions, from 2008 to 2018, and determine its association with the coverage of the Primary Health Care (Family Health Strategy and Basic Care models) in the Federal District.

**Methods:**

Ecological time series of mortality in Federal District elderly, from 2008 to 2018. The Poisson regression model was applied, considering as significant those with p<0.05, with a CI of 95%.

**Results:**

There were 70,503 deaths. There was a decrease in the risk of death of elders due to cardiovascular diseases and diabetes. Higher primary care coverage decreased the chance of death by sensitive conditions, both in Basic Care (OR: 0.994, CI: 0.990-0.998) and in the Family Health Strategy (OR: 0.997, CI: 0.995-0.999).

**Conclusions:**

Primary Care coverage was associated with a lower chance of death of the elderly due to Ambulatory Care Sensitive Conditions, especially in Basic Care.

## RESUMO

**Objetivos:**

Descrever os coeficientes de mortalidade entre idosos por condições sensíveis, de 2008 a 2018, e verificar a associação com a cobertura da Atenção Primária à Saúde (Modelo Estratégia Saúde da Família e Atenção Básica) no Distrito Federal.

**Métodos:**

Estudo ecológico tipo série temporal da mortalidade dos idosos no Distrito Federal, entre 2008 e 2018. Para análise das associações, aplicou se o modelo de regressão Poisson, sendo consideradas significantes as que apresentaram p<0,05, com IC de 95%.

**Resultados:**

Houve70.503 óbitos. Observou-se diminuição do risco de morrer dos idosos por doenças cardiovasculares e diabetes. A cobertura da Atenção Primária diminuiu a chance de morrer por condições sensíveis tanto na Atenção Básica (OR: 0,994, IC: 0,990-0,998) quanto na Estratégia Saúde da Família (OR: 0,997, IC: 0,995-0,999).

**Conclusões:**

A cobertura de Atenção Primária foi associada a menor chance de morrer dos idosos por condições sensíveis à Atenção Primária, sobretudo na Atenção Básica.

## RESUMEN

**Objetivo:**

Describir coeficientes de mortalidad entre ancianos por condiciones sensibles, de 2008 a 2018, y verificar relación con la cobertura de Atención Primaria de Salud (Modelo Estrategia Salud de la Familia y Atención Básica) en Distrito Federal.

**Métodos:**

Estudio ecológico tipo serie temporal de mortalidad de ancianos en Distrito Federal, entre 2008 y 2018. Para análisis de las relaciones, aplicado modelo de regresión Poisson, siendo consideradas significantes las que presentaron p<0,05, con IC de 95%.

**Resultados:**

Hubo70.503 óbitos. Observado disminución del riesgo de morir de ancianos por enfermedad cardiovasculares y diabetes. La cobertura de Atención Primaria disminuyó la probabilidad de morir por condiciones sensibles tanto en Atención Básica (OR: 0,994, IC: 0,990-0,998) mientras en Estrategia Salud de la Familia (OR: 0,997, IC: 0,995-0,999).

**Conclusiones:**

La cobertura de Atención Primaria fue relacionada la menor probabilidad de morir de ancianos por condiciones sensibles a la Atención Primaria, sobretodo en Atención Básica.

## INTRODUCTION

Health systems have been dealing with the challenge of providing adequate assistance to the elderly population. The Single Health System (SUS) is not well prepared to provide integral care to this population, since its growth is a recent phenomenon. Brazilian elderly suffer with asymmetric supply and demand of health services, which shows the shortcomings in the process of care^(1)^.

Primary Health Care (PHC) services are essential to provide care to the elderly, using models of care focused on therapeutic itineraries, adequate and organized according with their needs^(1-3)^. Withing the PHC, the Family Health Strategy (FHS) has been able to provide better care and decrease the mortality of this population^(4)^.

In this regard, studies have shown that an increased coverage of PHC contributes for more impactful actions to promote health and reduce mortality, especially in more vulnerable populations, such as the elderly^(5)^. A well-structured PHC can also reduce the hospitalizations in this group^(6)^. This age group requires hospitalization in health services due to multiple chronic conditions and functional and cognitive capacity loss^(7)^.

Therefore, an important indicator to monitor the quality and efficacy of PHC are hospitalizations by Ambulatory Care Sensitive Conditions (ACSC)^(8)^. These conditions have an epidemiological importance, as they can impact the mortality of the elderly. Thus, they are significant markers to analyze the quality of the care provided.

In 2008, the Ministry of Health published the hospitalization causes considered to be sensitive to PHC treatment, that is, that can be avoided, according to evidence, by chronic disease management, by the prevention of the deterioration of chronic conditions into acute events, and by other types of care^(7)^. This set of conditions can be managed safely in outpatient care when the diseases are detected and managed timely, avoiding deaths by these causes.

In the Federal District, investments have been made in the PHC, with a substantial increase in its coverage and the conversion of the traditional Basic Care (BC) model into the FHS model^(9)^. In this context, the present study is relevant as it analyzes the responses of the system in regard to the avoidable deaths of elderly persons. Therefore, nursing professionals have a relevant role in the elaboration of practices and, consequently, of a PHC model for the elderly.

## OBJECTIVE

To describe the mortality coefficients of elderly due to Ambulatory Care Sensitive Conditions, from 2008 to 2018, and to determine its association with the coverage of the Primary Health Care (Family Health Strategy and Basic Care models) in the Federal District.

## METHODS

### Ethical aspects

The study followed Resolution No. 466/12 from the National Council of Health (CNS). The process was approved by the Research Ethics Committee from the Faculty of Health Sciences of the Universidade de Brasília and by the Research Ethics Committee at the Fundação de Ensino e Pesquisa em Ciências da Saúde (FEPECS/SES/DF).

### Design, period, and place of study

This is an ecological study of the time series type, based on the mortality data of seniors citizens in the Federal District from 2008 to 2018, according with the initiative Statistical Analyses and Methods in the Published Literature(SAMPL)^(10)^.The Federal District is in the Brazilian highlands, in the Midwest of the country. Its population, in 2020, was estimated in 3,055,149 people, with 328,379 elderly, i.e. 10.7% of the population^(11)^. The Federal District, at the time of research, was undergoing a progressive increase of PHC coverage. In 2017, policies led to the conversion of PHC teams into FHS teams, leading to an expressive increase of the latter^(9)^.

### Population or sample; criteria for inclusion and exclusion.

The population of this study included all deaths in the Federal District of 60-year-old or older people from 2008 to 2018. The data from death certificates, as made available in the database of the Health Department of the Federal District, were analyzed. The data provided no identification and were obtained after the ethics committee approved the research and the relevant organ gave its authorization. At first, 86,903 deaths were extracted from the database. Data on elders who did not live in the Federal District were excluded, as well as certificates that did not include information on “cause of death”, “address”, and “date of birth”. After these exclusions, 70,503 certificates were left for analysis.

### Study protocol

Data collection included all seniors who were residents of the Federal District who passed away there, in the period mentioned above, according with the data from the Health Department. Data were extracted from the information system used by the Federal District Epidemiological Surveillance. Extraction took place in September 2019, after approval from the Ethics Committee.

The variables considered were: age group at time of death (60-69 y/o, 70-79 y/o, and 80 y/o or older); sex (male or female); marital status (single, married, stable union, separate/divorced, or unknown); race/color (white, black, brown, native, or unknown); primary cause of death; and year of death. The behavior of the coefficients of mortality by Ambulatory Care Sensitive Conditions was analyzed.

The deaths were classified according to the definition of Ambulatory Care Sensitive Conditions, according with Decree No. 221, from April 17, 2008^(12)^. The classification of causes of death in the study group used the tenth version of the International Classification of Diseases (ICD-10). The analysis of mortality curves was carried out for the main causes of death found.

The data about the coverage of Primary Health Care were found on the website about information and management of basic care, by the Ministry of Health (https://egestorab.saude.gov.br), using as a reference the month of December of each year in the historical series analyzed (2008-2018. The database provided aggregate PHC coverage, Basic Care coverage (BC), including the FHS model, in addition to the traditional BC model, equivalent and parameterized in regard to the population estimates. The database also provided the disaggregated coverage of FHS teams. The traditional BC model is divided in physician, gynecologist, and pediatrician, providing segmented care that is guided by the provision of services in the health care unit. The FHS model is based on teams formed by a physician, a nurse, a nursing technician, and a community health agent, whose objective is dealing with demands of the territory and provide guidance to the community^(13)^.

### Analysis of results and statistics

The statistical analysis included a descriptive analysis stage, considering the relative and absolute death rates. The calculation of death coefficients was estimated per 10 thousand people, supported by the projections of resident population numbers made by IBGE, the Brazilian geography and statistics institute (https://www.ibge.gov.br/estatisticas/sociais/populacao/9109-projecao-da-populacao.html). The risk of dying was calculated according to the relationship between the number of deaths per year by Ambulatory Care Sensitive Conditions and the number of elderly deaths in the same year. The measure was calculated for the group of general sensitive conditions and for the groups of interest for our analysis: cardiovascular conditions, pulmonary conditions, and diabetes. The process of construction of the database and the statistical analysis were carried out using IBM SPSS^®^ for Windows^®^, version 22.

A raw model for the analysis of the association of the variables with the outcome was used to verify the significance of the study. A significance level of 0.05 was established, with a confidence interval (CI) of 95%. For the multivariate analysis, Poisson’s regression model was used, and non-adjusted variables with p ≤ 0.20 were tested. Those that, at the end, presented p < 0.05, with a CI of 95%, were maintained. In the model, all variables mentioned were included, except the “home address of the elder”, as this information showed no statistical significance.

## RESULTS

This study analyzed 70,503 deaths from elders in the Federal District that took place from 2008 to 2018, as presented on Table 1. Most deaths were of women. Regarding their marital status, 38.4% of the elderly were married, and 31.3% were widowed. More than half (55.1%) of the dead were declared by their relatives as white, while 43.5% were declared to be black or brown. Deaths due to Ambulatory Care Sensitive Conditions represented 29.2% of cases.

**Table 1:** Distribution of deaths according with sociodemographic characteristics and Ambulatory Care Sensitive Conditions of the elderly, Federal District, Brazil, 2008 to 2018

Variables		No. of deaths	%
Sex	Female	35,402	50.2
	Male	35,101	49.8
Age group	From 60 to 69 years	18,993	26.9
	From 70 to 79 years	22,820	32.4
	80 yearsoldorolder	28,690	40.7
Marital Status	Single	12,467	17.7
	Married	27,105	38.4
	Widowed	22,052	31.3
	Separated/divorced	6,920	9.8
	Ignored	1,959	2.8
Race/color	White	38,816	55.1
	Black/Brown	30,699	43.5
	Asian	437	0.6
	Indigenous	43	0.1
	Ignored	508	0.7
Sensitive condition	Yes	20,606	29.2
	No	49,897	70.8

*Source: Databases of the Health Department of the Federal District.*

**Figure 1:**
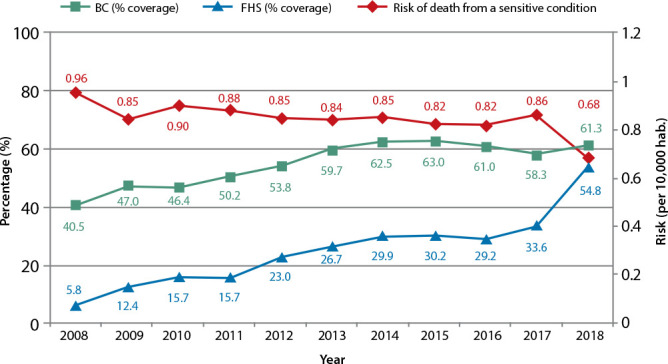
Risk of death due to Ambulatory Care Sensitive Conditions and percentage BC (Basic Care) and FHS (Family Health Strategy) coverage, according to the year of death, Federal District, Brazil, from 2008 to 2018

Figure 1 shows the BC coverage of the Federal District population, the FHS coverage, in separate, and the risk of death by Ambulatory Care Sensitive Conditions. There has been an expressive increase in the FHS coverage, indicating the importance of the model in the BC. The FHS represented, in 2008, 14% of the BC, having increased to 89% in 2018. It has also been observed that the risk of death due to sensitive conditions throughout the years has decreased. The years 2017 and 2018 stand out, with a decrease from 0.86 to 0.68.

**Figure 2:**
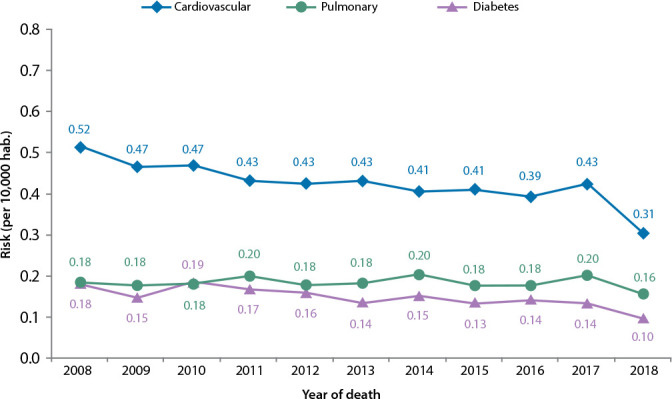
Risk of death due to Ambulatory Care Sensitive Conditions in the groups selected by this study, Federal District, Brazil, from 2008 to 2018

Figure 2 shows the behavior of mortality coefficients according to the most prevalent sensitive condition groups. The fall of the risk of dying from any of the sensitive conditions in the groups was higher from 2017 to 2018. The highest decrease was in the odds of death due to cardiovascular causes (sensitive to Primary Care).

Table 2 shows the factors associated with the likelihood of death of elders in the Federal District due to Ambulatory Care Sensitive Conditions, from 2008 to 2018, showing results that are in accordance with another study based on a hierarchical model^(14)^. In the non-adjusted model, males showed a higher chance of dying due to sensitive conditions. The age group was also related with the odds of death due to Ambulatory Care Sensitive Conditions, with a growing trend. Race/color, in the adjusted model, showed that black elderly were 6% more likely to die due to sensitive conditions. Regarding marital state, being married had a protective effect: married elderly were 15% less likely to die from sensible conditions when compared to single ones. Even separate/divorced elderly were 7.2% less likely to die from sensitive conditions than single elderly.

**Table 2:** Factors associated to deaths from Ambulatory Care Sensitive Conditions in the elderly population, Federal District, Brazil, 2008-2018

Independent variables	Total	Sensitive condition		Non-adjusted				Adjusted			
				OR^*^	CI (95%)^**^		*p*	OR	CI (95%)		*p*
		Yes	No		LI^***^	LS^****^			LI	LS	
Sex											
Female	35,402	10,953	24,449	1.181	1.143	1.220	<0.0001	-	-	-	-
Male (ref.)	35,101	9,653	25,448								
Age group											
From 60 to 69 years (ref.)	18,993	4,431	14,562								
From 70 to 79 years	22,820	6,615	16,205	1.342	1.284	1.402	<0.0001	1.298	1.237	1.361	<0.0001
80 years old or older	28,690	9,560	19,130	1.642	1.575	1.712	<0.0001	1.534	1.462	1.608	<0.0001
Race/Color											
Not black (ref.)	39,804	11,333	28,471								
Black	30,699	9,273	21,426	1.087	1.052	1.123	<0.0001	1.059	1.021	1.099	0.002
Marital status											
Single (ref.)	12,467	3,923	8,544								
Married	27,105	7,045	20,060	0.765	0.730	0.801	<0.0001	0.852	0.809	0.897	<0.0001
Widowed	22,052	7,209	14,843	1.058	1.009	1.109	0.019	-	-	-	-
Separated/Divorced	6,920	1,858	5,062	0.799	0.749	0.853	<0.0001	0.928	0.865	0.995	0.037
Education											
None	13,848	4,912	8,936	1.856	1.767	1.949	<0.0001	1.638	1.553	1.727	<0.0001
Up to 7 years	32,100	9,631	22,469	1.447	1.387	1.509	<0.0001	1.370	1.311	1.431	<0.0001
8 years or more (ref.)	18,177	4,154	14,023								
Basic Care Coverage (%)	-	-	-	0.992	0.990	0.994	<0.0001	0.994	0.990	0.998	0.005
Family Health Strategy Coverage (%)	-	-	-	0.995	0.994	0.996	<0.0001	0.997	0.995	0.999	0.036

* OR: Odds Ratio

** CI: Confidence interval

*** LL: Lower Limit

**** UL Upper Limit.

A lower educational level was associated with a higher chance for death from sensitive conditions, presenting a growing trend. Regarding the Primary Care coverage, both BC coverage and exclusive ESF coverage had a protective effect. A 10% increase in the BC coverage was associated to a 6% diminution in the odds of death of elderly due to Ambulatory Care Sensitive Conditions. The same increase in ESF coverage led to a 3% decrease in the likelihood of death from these causes.

## DISCUSSION

Death by Ambulatory Care Sensitive Conditions progressively decreased in the period of 2008 to 2018, in the Federal District. Other studies presented similar results^(15)^, as one carried out in Santa Catarina from 2008 to 2015^(16)^, and another from Rio Grande do Sul, carried out from 2008 to 2016^(17)^. However, in Finland, the death of elders due to Ambulatory Care Sensitive Conditions increased, when all causes were compared^(18)^.

Elderly mortality due to Ambulatory Care Sensitive Conditions is influenced by both coverage and access^(19-21)^, but the supply of these services is still a limiting factor^(20)^, especially for populations who have to face geographic obstacles. In the PHC, elders receive longitudinal follow up and are embraced and evaluated, their main diseases and weaknesses are identified and, in this context, a care plan that can reduce mortality is proposed^(22)^. Therefore, the likelihood of death of the researched seniors decreased due to the increase of PHC coverage.

In the Federal District, local policies were created to increase PHC coverage, including the increase in traditional BC, which was the prevalent model before the period of the study, and that of the FHS. In this regard, a policy from 2017 was especially relevant, as it considerably increased the number of FHS teams. This policy allowed, in addition to the increase in coverage, a substantial increase in the number of houses assisted. It was followed by investments, professional capacitation, and monitoring^(9,23)^. The population under study showed, in the time series, a decrease in the likelihood of death from Ambulatory Care Sensitive Conditions.

This study found a decrease in the odds of death due to cardiovascular causes. This group of diseases is among the main death causes among the elderly^(24)^. A national study showed that, from 2000 to 2011, the number of hospitalizations due to this group of diseases decreased. This was explained by improvements to the access and quality of PHC actions^(25)^. In this context, PHC has an important role in the control and prevention of the negative outcomes of this group of diseases^(26)^. There are many factors involved in dealing with chronic diseases, and their treatment depends on an increased access to health services, on the availability of medication, and on diagnostic instruments^(27)^. The role of physical activity to reduce the mortality from cardiovascular diseases stand out, as this is a simple, cheap, and replicable Primary Care measure^(28)^.

Pulmonary diseases are also very prevalent, when compared to other groups of causes, and are related with ACSCs. This study showed an oscillation in the risk of death from these causes. In Brazil, pulmonary diseases, especially pneumonias, presented a significant increase, leading to the death among elders. Nonetheless, there was a decrease in deaths from chronic obstructive lung disease^(29)^. The vaccination program is in the PHC, and elders are a priority group, which has an effect on their mortality, especially due to the H1N1 vaccine^(30)^. In addition to vaccination, other risk factors for pulmonary diseases are handled by Primary Care, such as obesity, smoking, and problems to swallow^(31)^. In this context, the FHS team has an important role in the reduction of mortality from pulmonary causes, especially in underprivileged communities^(32)^.

The risk of death from diabetes also decreased in the period analyzed. The assistance of Primary Care workers is relevant in this disease^(33)^, especially that of nursing workers, as it can improve diagnosis and clinical management, contributing to lower mortality^(34)^. Furthermore, positive experiences and workers who have a good relationship with patients showed better results in the control of type-2 diabetes^(35)^. Uncontrolled diabetes is related with a higher mortality and an increase in the infections and hospitalizations^(36)^. In the scope of PHC, educational interventions help raise awareness about the treatment, improving the control of disease and increasing the potential to reduce deaths^(37)^. Counseling about life habits substantially contributes for deaths in diabetic patients^(38)^.

### Study limitations

Limitations of this study include the fact that mortality studies can be impacted when the causes of death are not properly filled in in the death certificates. Due to the methodological nature of the study, there may have been an ecological bias in all relationships analyzed. The health system of the Federal District is often accessed by people from neighboring cities. In this process, patients occasionally inform inexact local addresses to guarantee they will receive attention. As a result, some of the deaths included in the study may correspond to elders who did not live in the Federal District. After the exclusion criteria were analyzed, more than 16 thousand deaths were removed from the study, which can limit the analysis of the results. It also stands out that the policy to increase FHS coverage mentioned was put into effect at the end of the historical series; therefore, the decrease in deaths by chronic diseases might have been influenced by other factors that this study did not measure.

### Contributions to the fields of Nursing, Health, or Public Policy

Understanding the mortality of elders due to ACSC in the different Primary Care models is extremely relevant for nursing workers, considering the potential positive effects of health prevention and promotion activities carried out by them and their impact on Ambulatory Care Sensitive Conditions. This study showed that the PHC has a protective effect in regard to the death of elders, showing the need for nurses to invest in this model of care by providing continued care to this population.

## CONCLUSIONS

There was a decrease in the risk of death of elderly due to Ambulatory Care Sensitive Conditions (cardiovascular diseases and diabetes) in the Federal District. PHC coverage had a positive association with lower chance of death by sensitive conditions in elderly. A 10% increase in BC represented a 6% decrease in the chance of death. The same increase in the FHS coverage was associated with a 3% reduction in the odds of death. These results suggest the need to continue qualifying nursing care to the elderly, especially considering the advanced practices in the primary level of health care. Furthermore, this study shows the importance of robust public policies in regard to quality PHC with an adequately extended coverage.
